# Antimicrobial Prophylaxis and Modifications of the Gut Microbiota in Children with Cancer

**DOI:** 10.3390/antibiotics10020152

**Published:** 2021-02-03

**Authors:** Gianluca Bossù, Riccardo Di Sario, Alberto Argentiero, Susanna Esposito

**Affiliations:** Pediatric Clinic, Pietro Barilla Children’s Hospital, University of Parma, 43126 Parma, Italy; gianlubollis@gmail.com (G.B.); rickds93@gmail.com (R.D.S.); alberto.argentiero@unipr.it (A.A.)

**Keywords:** antibiotic, antimicrobials, cancer, children, dysbiosis, microbiota

## Abstract

In children with cancer, chemotherapy can produce cytotoxic effects, resulting in immunosuppression and an augmented risk of febrile neutropenia and bloodstream infections. This has led to widespread use of antibiotic prophylaxis which, combined with intensive chemotherapy treatment, could have a long-term effect on the gastrointestinal microbiome. In this review, we aimed to analyze the current literature about the widespread use of antibiotic prophylaxis in children experiencing infectious complications induced by chemotherapy and its effects on the gut microbiome. Our review of the literature shows that antimicrobial prophylaxis in children with cancer is still a trending topic and, at the moment, there are not enough data to define universal guidelines. Children with cancer experience long and painful medical treatments and side effects, which are associated with great economic and social burdens, important psychological consequences, and dysbiosis induced by antibiotics and also by chemotherapy. Considering the importance of a healthy gut microbiota, studies are needed to understand the impact of dysbiosis in response to therapy in these children and to define how to modulate the microbiome to favor a positive therapeutic outcome.

## 1. Introduction

Cancer diagnoses in patients younger than 20 years old are rare, representing only approximately 1% of all new cancer cases every year, and the survival rate increased to 84.8% from 2008 to 2013 in all age groups 0–19 years [1]. The epidemiology of cancer in children differs from that in adulthood: lymphohematopoietic cancers account for approximately 40%, central nervous system (CNS) cancers account for approximately 30%, and embryonal tumors and sarcomas account for approximately 10% of cases. The most frequent cancer in childhood, in particular, is leukemia (acute lymphoblastic leukemia [ALL] and acute myeloid leukemia [AML]), with ALL accounting for 26% of all cancers in children up to 14 years of age and 75% of all pediatric leukemia cases [2]. Children with cancer undergo a treatment protocol that may include chemotherapy, radiotherapy (RT), surgery, or hematopoietic cell transplantation (HCT) alone or in combination depending on the type of cancer, its location, and its stage.

The use of some chemotherapeutics can produce cytotoxic effects, resulting in immunosuppression and an augmented risk of febrile neutropenia (i.e., the total number of blood neutrophils ≤ 500/mm^3^) and bloodstream infections [2]. This has led to widespread use of antibiotic prophylaxis, which, combined with intensive chemotherapy treatment, could have a long-term effect on the gastrointestinal microbiome [3]. The effects of this alteration have been studied but remain mostly unknown; it has been proven that this change leads to colonization by opportunistic pathogens, impairs the gastrointestinal barrier, increases vulnerability to *Clostridium difficile* infections [3], and is also linked to the development of a variety of diseases. In this review, we aimed to analyze the current literature about the widespread use of antibiotic prophylaxis in children experiencing infectious complications induced by chemotherapy and its effects on the gut microbiome. A further purpose was to show the current known effects of alterations of the microbiome on children’s responses to chemotherapy and their future health outcomes. Eventually, we wanted to focus briefly on probiotics, their actual use, and their possible more routine application in clinical practice. PubMed was used to search for studies published mainly in the last 15 years using the key words “cancer”, “microbiome” or “microbiota”, “antibiotic prophylaxis”, and “children” or “pediatric”. More than 150 articles were found, but only those written in English were taken into consideration.

## 2. Overview of Antibiotic Usage in Children with Cancer

### 2.1. Infectious Complications in Children with Cancer

Infectious complications are among the most common and life-threatening complications; they are associated with significant morbidity and mortality and lead to treatment delays and dose reductions of chemotherapy [4]. Their development in children with cancer mainly occurs during periods of neutropenia [5,6], and is favored by the presence of skin and mucosal damage, central venous catheters, or the use of immunosuppressant drugs [5].

Fever is the first and most frequent symptom of bacterial infection, especially during periods of neutropenia. Castagnola et al. carried out a three-year observational study showing that the highest frequency of neutropenic periods with primary febrile episodes was observed after autologous HCT (58%), during induction treatment for ALL or non-Hodgkin lymphoma (48%), and after allogeneic HCT (44%) [7]. In a study conducted by Gil et al., 92.3% of patients diagnosed with cancer developed infectious complications after high-dose chemotherapy or HCT [8]. A more recent observational study by Zawitkowska et al., focusing on children with newly diagnosed ALL, showed that 53.2% of them had a microbiologically documented bacterial infection during chemotherapy.

### 2.2. Etiology of Bacterial Infections

The most common infections during periods of neutropenia are bacterial infections, and there has been a clear shift in the type of organisms involved during the past three decades [4]: until the beginning of the 1980s, gram-negative rods (particularly *Escherichia coli, Klebsiella* spp., and *Pseudomonas aeruginosa*) were the most frequent causes of bacterial infections in cancer patients [5,6,7,9], but recently, an increase in the frequency of infections caused by gram-positive organisms in these patients occurred [9]. Gram-negative bacteria are the main causes of bloodstream infections, whereas gram-positive bacteria cause mainly central venous catheter (CVC)-related infectious complications. However, infections caused by gram-negative bacteria are associated with higher morbidity and remain the most common cause of mortality during periods of myelosuppression [4,10,11].

## 3. Antibiotic Prophylaxis

The severity of bacterial infections during periods of neutropenia in children with cancer makes essential the appropriate use of broad-spectrum antibiotics to resolve this kind of complication and to reduce associated morbidity and mortality. Many different approaches have been discussed in recent years, particularly concerning the correct classes of antibiotics to use on these occasions and whether to prevent infectious complications with systemic antibiotic prophylaxis or to treat them when microbiologically documented [12,13]. In the current literature, there is a lack of clinical guidelines concerning the routine use of antibacterial prophylaxis. A recent study by Lehrnbecher et al. reviewed the works on this argument to develop a guideline for the administration of antibiotic prophylaxis in pediatric clinical practice, proving that there are not enough studies supporting the routine use of antibiotics in children with cancer or HCT recipients because the benefits of prophylaxis are balanced by its unknown and potential impacts and resistance [12]. However, antimicrobial prophylaxis for neutropenic patients undergoing cytotoxic therapy reduces mortality, as supported by a meta-analysis published by Gafter-Gvili et al. [13], showing the importance of appropriate administration and selection of the correct class of antibiotic.

### 3.1. Beta-Lactam Antibiotics

Beta-lactam antibiotics work by inhibiting cell wall biosynthesis in the bacterial organism and are the most widely used group of antibiotics. At first, beta-lactam antibiotics were mainly active only against gram-positive bacteria, yet the recent development of broad-spectrum beta-lactam antibiotics active against various gram-negative organisms has increased their usefulness [5]. The cephalosporin class and amoxicillin/clavulanate have been widely studied and used, as proven by Castagnola et al., who employed prophylaxis with amoxicillin/clavulanate at a dose of 25 mg every 12 h and showed a reduction in febrile episodes [14]. Feng et al. studied the incidence of infection-related fever in children with AML and the outcome of prophylactic use of vancomycin/cefepime or piperacillin/tazobactam versus a control group with no antibiotic prophylaxis: the result was a significant reduction in infection-related fever in those children undergoing antibiotic prophylaxis during periods of chemotherapy-induced neutropenia [15]. However, in the last decade there has been a worldwide increase in multidrug-resistant (MDR) bacteria, making the oral therapy approach with beta-lactams ineffective in many cases [16,17,18,19].

### 3.2. Fluoroquinolones (FQLs)

FQLs interfere with DNA replication by preventing bacterial DNA from unwinding and duplicating. They are effective against both gram-negative and gram-positive bacteria [5]. FQLs were formerly used in the prophylaxis of adult patients with cancer because of their great efficacy, as proven by two large studies conducted in 2005 [20,21]. Since then, there has been growing interest concerning the routine use of FQLs in antibiotic prophylaxis because they have a broad antibacterial spectrum, good bioavailability and bactericidal activity, are well tolerated, and do not have any myelosuppressive effects. Indeed, a recent review by Lehrnbecher showed that FQL prophylaxis significantly reduced bacteremia, fever, and neutropenia; it was not significantly associated with *C. difficile* infection, invasive fungal disease, or muscle–skeletal toxicities, but it was significantly associated with more FQL resistance in bacteremia isolates [12]. One of the first works on this topic was performed by Cruciani et al., who studied the effects of norfloxacin compared with that of trimethoprim-sulfamethoxazole (TMP-SMX) in 44 neutropenic children with various malignancies, proving that FQL was superior in preventing febrile episodes, but the mean number of febrile days was similar in the two groups [5,22].

Recent studies focused on ciprofloxacin, whose effects have been widely studied in children with cancer, and levofloxacin, which seems to be the preferred agent if antibacterial prophylaxis is planned [12]. Alexander et al. studied the effects of prophylaxis with levofloxacin at a prophylactic dosage of 10 mg/kg twice daily (in children aged six months to five years) or 10 mg/kg once daily (for children aged more than five years) in 624 patients with AML, with relapsed ALL, or who underwent HCT, proving that among children with acute leukemia receiving intensive chemotherapy, the administration of levofloxacin prophylaxis compared with no prophylaxis resulted in a significant reduction in bacteremia; however, there was no significant reduction in bacteremia for levofloxacin prophylaxis among children undergoing HCT [23]. Ciprofloxacin also has a good efficacy profile, as proven by Laoprasopwattana et al., who compared his effects with a placebo group in 95 patients with lymphoma and ALL and observed a reduction in fever and bacteremia in those receiving FQL [24] during induction but not during the consolidation phase. Widjajanto et al. conducted a similar study in 110 children with ALL who were undergoing induction treatment and compared the effects of prophylaxis with ciprofloxacin with those of placebo; the result was disappointing, with a greater risk of fever and sepsis and increased mortality among those who received ciprofloxacin [25]. The dosage of ciprofloxacin has varied among the studies: Yousef et al. used ciprofloxacin at a prophylactic dosage of 25 mg/kg/day [26], Laoprasopwattana at a dosage of 20 mg/kg/day [24], Choeyprasert at 20–30 mg/kg/day in two divided doses in combination with penicillin sodium V 25–50 mg/kg/day in four doses [27], while Al Omar et al. administered it at a dosage of 10 mg/kg/day divided into two doses every 12 h [28].

### 3.3. Trimethoprim-Sulfamethoxazole (TMP-SMX)

TMP-SMX, also known as co-trimoxazole, is a combination of two antimicrobial agents that act synergistically against a wide variety of bacteria. The two components, TMP and SMX, work sequentially to inhibit enzyme systems involved in the bacterial synthesis of tetrahydrofolic acid [5]. TMP-SMX prophylaxis is a valid alternative to FQLs that reduces bacteremia and infection-related mortality, but it increases resistance in bacteremia isolates [12]. It has broad-spectrum activity, including gram-positive bacteria, gram-negative bacteria, Nocardia, and *Pneumocystis jirovecii.* Its efficacy profile was supported by some studies conducted by Goorin et al. [29], which showed fewer fever episodes in children treated with TMP-SMX than in children treated with the placebo [29], and by Kovatch et al., which found a reduction in fever and bacteremia related to its use [30]. However, these positive aspects are balanced by a series of drawbacks, including the development of hypersensitivity, breakthrough infections due to resistant gram-negative and gram-positive pathogens, fungal infections, and *C. difficile* colitis, as evidenced by Gualtieri et al. [29,30,31]. For the appropriate prophylactic dose, Schroder et al. used it at a dosage of 10–30 mg/kg/day [32], Cullen et al. employed a fixed dosage of 20 mg/kg/day [21], Chastagner et al. a prophylactic dosage of 25 mg/kg/day every two days [33], while Al Omar et al. used TMP-SMX at a dosage of 2.5 mg/kg/day in combination with ciprofloxacin for two consecutive days per week [28].

In Table 1, we report the main studies on antibiotic prophylaxis performed by the authors cited above, who administered different agents at different dosages, showing that there are no official and standard guidelines for antibiotic prophylaxis in children with cancer. As demonstrated, antibiotic prophylaxis in these patients may have a fundamental role in preventing and reducing infection-related mortality in children with a high risk of febrile neutropenia and infectious complications, but more studies are required to define the best treatment, to ensure a correct balance of positive and adverse effects, to show the feasibility of all the different molecules available, and to establish a widely accepted guideline [28,34].

## 4. Dysbiosis and Cancer in Children

The human gut microbiota consists of thousands of different species, each sharing a symbiotic relationship with the human host [35]. It appears that each person has his or her own individual suite of microbial strains [36,37]; this bacterial “panel” is acquired in the early stages of life [38] and can remain unaltered or go through different transitions [39,40]. Dysbiosis, an imbalance in microbial taxa, has been implicated in various aspects of human health. One of the main factors that can disrupt a healthy gut microbiota is antibiotic exposure [41]: even short-term antibiotic usage has been linked to the loss of certain taxa (i.e., a reduction in the diversity of *Firmicutes* and *Bacteroidetes*, growth of the family *Enterobacteriaceae*) [42,43], impairment of the gastrointestinal barrier [44], and increased vulnerability to *Clostridium difficile* [45] and other vancomycin-resistant *Enterococci* [46]. Even within days of the administration of antibiotics, a significant upregulation of resistance genes has been shown [41]. In children, it has been shown that a disrupted intestinal microbiota is linked to the development of a range of diseases (such as inflammatory bowel diseases [47], Kawasaki syndrome [48], asthma [49], autism [50,51,52]), and most importantly, dysbiosis itself plays an important role in cancerogenesis, alongside environmental and genetic factors [53,54]. Since antibiotics are largely used to prevent infectious complications in neutropenic children undergoing chemotherapy [55] and represent some of the most frequently prescribed drugs in pediatric patients [56,57], a better understanding of the dynamic interaction between gut microbiota dysbiosis and cancer pathogenesis may be helpful to improve the standard of care in children with cancer. On the other hand, chemotherapy itself can further worsen the microbiota composition.

### 4.1. Gut Microbiome Alterations in Children with Acute Leukemia

Acute lymphoblastic (ALL) and myeloid (AML) leukemia are the most frequent childhood blood malignancies [58]; therefore, alterations in the microbiota at the time of diagnosis and during chemotherapy have been analyzed mostly in children with leukemia. It has been shown that in both ALL and AML patients, the amount of bacterial flora is reduced in comparison with that in healthy controls: specifically, a significant decrease in *Bifidobacteria, Lactobacillus*, and *E. coli* has been found in children with leukemia [59,60]. Rajagopala et al. [3] collected stool samples from 51 children, both pediatric and adolescent patients with ALL and healthy siblings, to identify possible variations in the gut microbiota before and during chemotherapeutic treatment; *Bacteroides, Prevotella*, and *Faecalibacterium* were found in both groups at the time of diagnosis, but the overall microbial diversity of the ALL group was lower than that of the healthy sibling group (*p* < 0.01). Microbial diversity was not significantly different at the end of chemotherapy but increased significantly at different visits after the end of chemotherapy (*p* < 0.01). Van Vliet et al. [60] collected stool samples from pediatric patients with AML who were undergoing chemotherapy and receiving antimicrobial prophylaxis against gram-negative bacteria and fungi (oral colistin, neomycin, and amphotericin B with ciprofloxacin and itraconazole), *viridans* group streptococci (oral pheneticillin), and *Pneumocystis jirovecii* (oral cotrimoxazole); they found a large decrease in anaerobic bacteria, compensated by an increase in potential pathogenic *Enterococci*. These studies show that antibiotic-induced dysbiosis could be dangerous in critically ill patients but is not solely responsible for such disruption of the gut microbiota; the interaction between chemotherapy and different factors (genetics, diet) [61] in shaping dysbiosis may be the key to obtaining a better understanding of the dynamics underlying cancerogenesis.

### 4.2. Microbiota and the Patient’s Outcome: Infections, Adverse Effects, and Response to Treatment

Infections are a common and dangerous complication in children with cancer [4,62]. In addition to bloodstream infections (generally caused by gram-negative bacteria [63]), CVC-related infectious complications are also a relevant cause of morbidity. It has been estimated that 14–51% of CVCs implanted in children with malignancies may be complicated by bacteremia [64]. Galloway et al. [65] collected buccal and fecal specimens twice weekly from 34 AML patients undergoing induction chemotherapy to define a possible link between antimicrobial composition and infection outcomes. They observed that a low baseline α-diversity in stool samples was associated with the development of infections during chemotherapy; patients before leukemia treatment had a wide range of α-diversity, indicating that the factors causing this variability are numerous and may include previous antibiotic exposure, diet, and genetics. Interestingly, it was noted that higher antibiotic exposure during induction chemotherapy was significantly associated with an increased risk of infection after treatment.

While early administration of broad-spectrum antibiotics has been shown to reduce mortality, there is some evidence that prophylactic antibiotic regimens may alter the human microbiota, thus inducing antibacterial resistance and the proliferation of MDR bacteria [62]. Two similar nonrandomized studies [66,67] evaluated blood cultures from pediatric cancer patients presenting with febrile neutropenia; in both studies, some patients underwent antibiotic prophylaxis with ciprofloxacin (a small portion of the patients in one study were not treated, but interestingly, all adult patients from the same center received FQL prophylaxis). The results were similar: in children receiving ciprofloxacin, gram-negative bacteria found in blood cultures had increased rates of resistance towards multiple antibiotics. Even children who did not receive ciprofloxacin but were hospitalized in the same institution where adults underwent FQL prophylaxis had this pattern of resistance, suggesting that factors in addition to antibiotic exposure must be involved. TMP-SMX prophylaxis does not seem to cause these alterations, but fewer data are available [68].

A frequent gastrointestinal complication in children with cancer is diarrhea, which can seriously impair the patient’s quality of life as it leads to malnutrition and fatigue [69,70]. While chemotherapy and radiation are proven risk factors for mucositis and therefore acute diarrhea [71,72], the gut microbiota has recently been proposed as a potential factor in the onset of this complication [73,74,75,76,77,78,79,80,81,82,83]. In a study by Manichanh et al. [73], fecal samples from 10 patients undergoing radiotherapy for abdominal tumors were collected at different times during treatment; six of them suffered from diarrhea and showed a progressive reduction in microbial diversity, while the other four had a “stable” gut microbiota compared with that of a healthy control group. Similar results have been seen in post-chemotherapy diarrhea, with the gut microbiota changing towards *Escherichia coli* and *Staphylococcus* domination with decreases in *lactobacilli, Bifidobacteria, Bacteroides*, and *Enterococci* [74]. Even if very few data are available concerning the role of microbiota in pediatric patients with cancer presenting diarrhea [60], probiotics are now taken into consideration in different studies as a possible tool to prevent chemotherapy- and radiotherapy-related mucositis [75]. Finally, some authors have suggested a potential interaction between the gut microbiota and anticancer drugs, thus influencing antineoplastic treatment. In murine models, there is increasing evidence of this particular interaction. Iida et al. [76] showed that mice with subcutaneous EL4 lymphoma who received an antibiotic cocktail prior to oxaliplatin treatment had a significant reduction in cancer regression and survivability when compared with an “antibiotic-free” control group. These findings are replicated in another interesting study by Gui et al. [77], in which animals with lung cancer receiving a combination of cisplatin and antibiotics had a decreased survival rate in comparison to mice treated with cisplatin alone. On the other hand, the administration of cisplatin and *Lactobacillus* bacteria improved the response to therapy.

The mechanism that may underlie the relationship between gut microbiota and chemotherapy is summarized in Table 2. Several studies on this topic are still ongoing, and with novel drugs coming out every year and the growing usage of monoclonal antibodies in different stages of cancer, many more relationships are yet to be discovered.

### 4.3. The Gut Microbiota Plays a Key Role as Trigger for Gut Graft Versus Host Disease in the Context of Hematopoietic Stem Cell Transplantation (HCT)

When cancer or cancer treatments destroy the stem cells, HCT may be the best treatment option. The possible link between gut microbial disruption and HCT has been widely studied, and it is currently clear that dysbiosis is one of the many actors in the genesis of graft versus host disease (GvHD), a potentially lethal complication of HCT.

The first studies on this topic were performed in the 1970s [84,85], when analyses in mice showed a lower incidence of gut GvhD in germ-free animals. Since then, much progress has been made, especially thanks to the use of next-generation sequencing. The work of Holler et al. [86] is particularly relevant: stool specimens from 31 patients receiving HCT were analyzed before and after the procedure through next-generation sequencing. Before HCT, all patients showed a similar balance of commensal bacteria. After HCT, a shift towards *Enterococcus* domination was observed, which was particularly evident in patients under antibiotic treatment who developed gut GvHD. This was one of the first studies to provide evidence that alteration of the microbiota by HCT-related procedures (such as antibiotic exposure) is directly linked to GvHD, as confirmed in more recent analyses [87,88,89,90]. Italian researchers have been particularly active in defining how microbiota dysbiosis impacts pediatric patients undergoing HCT. A first work published in 2015 [91] showed that changes seen in adult patients also occurred in children. Pre- and post-HCT samples were collected from 26 pediatric patients undergoing HCT and analyzed with next-generation sequencing to define possible variations in microbiota structure. Interestingly, children who suffered from GvHD had specific gut microbiota signatures after HCT: overgrowth of *Enterococcus* and *Clostridiales*, decreases in *Faecalibacterium* and *Ruminococcus*, and a substantial decrease in *Bacteroides*, a group of bacteria associated with the production of propionate, which might have the ability to activate T helper type 2 cells [92,93]. Moreover, Biagi et al. [94] tried to determine with more accuracy if these differences in the gut microbiota population were already established before HCT. Stool specimens from 36 pediatric patients undergoing HCT were collected before transplantation, at the time of engraftment and after 30 days. Next-generation sequencing showed that children who suffered from GvHD had dysbiosis before HCT (a lower number of Blautia, a higher abundance of *Fusobacterium*, and decreased overall diversity), thus implying that the gut microbiota may be a tool to stratify GvhD risk. Finally, in a recent study by d’Amico et al. [95], the gut resistome, meaning the pattern of antibiotic resistance derived from the gut microbiome, was taken into consideration. A comparison was made between 8 pediatric patients undergoing HCT and 10 healthy adults. Interestingly, an amplification of the resistome was noted, especially in four patients who developed acute GvHD, even though antibiotics were not routinely administered in the course of transplantation (aminoglycosides, macrolides, and tetracyclines). All this evidence suggests that microbiota and HCT outcomes are deeply intertwined, even in pediatric patients, in whom the microbiota is often still taking shape and in some sense immature. Prebiotics, probiotics, or fecal microbiota transplantations might be fundamental tools for preventing major damage associated with this procedure.

### 4.4. Efficacy of Probiotics in Children with Cancer

Probiotics are live microorganisms, which, when administered in adequate amounts, confer a health benefit on the host [96,97]. Probiotic supplementation has been widely studied in children presenting various health conditions [98,99,100,101,102,103,104], whereas there have been very few studies regarding gut microbiota dysbiosis in pediatric patients with cancer. The first one was published in 1980 [105] and was also one of the first works that examined the concept of intestinal decontamination, that is, a prophylactic strategy that consists of the administration of antimicrobials with limited anaerobicidal activity in order to reduce the burden of aerobic gram-negative bacteria and/or yeast in the intestinal tract and so prevent infections caused by these organisms. In a group of 68 children with leukemia and solid tumors, 35 neutropenic episodes in 33 children were treated with framycetin, colymycin, nystatin, and metronidazole, while the other 35 episodes in the remaining 35 children were cured with TMP-SMX and *Lactobacillus* preparations [105]. Even if there was no significant difference in the incidence of infections during the period of neutropenia, the second group had better tolerance to the medication, so the authors concluded that a TMP-SMX and *Lactobacillus* preparation may improve quality of life in neutropenic children and is also relatively inexpensive [105]. Finally, Wada et al. [106] demonstrated that children with cancer receiving *Bifidobacterium breve* strain Yakult had decreased levels of *Enterobacteriaceae* in their stool and, more importantly, children assigned to the probiotic group had fewer febrile episodes (0.5 ± 0.62, 95% confidence interval [CI]: 0.21–0.79 and 1.06 ± 1.80, 95% CI: 0.19–1.93) than did children assigned to the placebo group (0.95 ± 0.79, 95% CI: 0.62–1.28 and 3.00 ± 3.84, 95% CI: 1.39–4.61), thus indicating lower usage of antibiotics. Nevertheless, there is much more work to be done, especially since some researchers have shown concerns about the possibility of administering living microorganisms to patients with compromised immunity and gut defenses after reports of sepsis caused by probiotic pathogens [107,108,109]. Further studies are needed to define the feasibility and correct duration of probiotic administration, when it should be suspended, and if it has a real impact in preventing major gut comorbidities in children with solid or hematological malignancies.

## 5. Conclusions

Our review of the literature shows that antimicrobial prophylaxis in children with cancer is still a trending topic and, at the moment, there are not enough data to define universal guidelines. The reasons for this challenge are numerous. First, a child is an “evolving” being: the immune system changes rapidly in pediatric age and drastically over time, as does its response to certain drugs. Table 3 summarizes the effects of different antibiotics on intestinal microbiota. Finding a standard prophylaxis for children of all ages might be hard, especially because most studies take into account the use of FQLs, drugs not registered for children. Moreover, different factors come into play when discussing antibiotic prophylaxis. Not only is the right dosage fundamental but also the feasibility of the prophylaxis itself is an element that needs to be taken into consideration in future studies; when we discuss pediatric patients, the number of administrations and the palatability [110] of a drug are key points, as these are among the major factors affecting pediatric compliance.

In addition to defining the optimal antibiotics needed for prophylaxis, there is still uncertainty about patients who do not need treatment. With antimicrobial resistance being a global health security threat [111], more accurate antibiotic prescriptions should be implemented, especially in patients with cancer, to prevent serious complications and, as we have described in our review, disruption of the intestinal microbiota, an emerging player in maintaining human health. Many researchers around the world have focused on obtaining a better understanding of the influence of the gut microbiota in different health conditions, but only in recent years has the idea of modulating the microbiome to modify the outcome of hematological/oncological diseases started to attract attention. Novel strategies for preventing antibiotic-mediated dysbiosis have been proposed: some authors have suggested the use of beta lactamase enzymes for the degradation of antibiotics in the gut [112], while others proposed that changing nutritional strategies in oncological patients, such as promoting enteral nutrition rather than parenteral nutrition or the administration of specific molecules (probiotics), might be the key to preventing gut complications [113]. Another approach for the prevention of dysbiosis is the possibility of intervening in the nutritional state of the patients. Children undergoing chemotherapy exhibit several variations in their body composition, with a higher percentage of fat mass and a lower body cell index [114] or a general reduction in bone density [115]. Probiotics may be helpful in these patients since some of them can regulate protein absorption and utilization [116,117].

In conclusion, children with cancer experience long and painful medical treatments and side effects, which are associated with great economic and social burdens, important psychological consequences, and dysbiosis induced by antibiotics and also by chemotherapy [118,119,120]. Considering that a healthy gut microbiota keeps the gut epithelium intact, studies are needed to understand the impact of dysbiosis in response to therapy in children with cancer and to define how to modulate the microbiome to favor a positive outcome.

## Figures and Tables

**Table 1:** Main studies regarding antibiotic administration in children with cancer.

Author, Year	Criteria for Prophylaxis	Antibiotics Employed
Cecinati, 2013	Children with cancer and probably long-lasting neutropenia, in accordance to the chemotherapics employed	− TMP-SMX → 10–30 mg/kg/die or 20 mg/kg/die − CPF → 25 mg/kg/die or 20 mg/kg/die − Amoxicillin + clavulanic acid → 25 mg twice daily for 15 days maximum
Chastagner, 2018	Patients with AML or ALL in order to prevent infections related to mortality	− Oral TMP-SMX 25 mg/kg/die every two days
Choeyprasert, 2017	To all HCT recipients on the day on which conditioning regimens started, until engraftment, and discontinuation was indicated when the patients developed fever, clinically documented infection or suspected infection	− Oral Ciprofloxacin 20–30 mg/kg/die in two divided doses + Penicillin V sodium 25–50 mg/kg/die in four doses
Al Omar, 2017	To each CT course when the ANC was ≤1000 cells/mm^3^ and continued until AMC was ≥100 cells/mm^3^ postnadir	− Ciprofloxacin → 10 mg/kg/die every 12 h − Cotrimoxazole → 2.5 mg/kg/die every 12 h for two or consecutive days per week
Alexander, 2018	To all patients aged 6 months to 21 years with acute leukemia (any AML or relapsed ALL) or HCT recipients	− Levofloxacin → 6 months to 5 years: 10 mg/kg twice daily; >5 years: 10 mg/kg once daily

**Table 2:** Main studies on the gut microbiota and chemotherapy dynamics.

Author, Year	Finding
Huang [78], 2001	Certain bacteria reside in the tumor tissue and can directly modulate chemotherapy by producing nucleoside analogue-catabolizing enzymes, which can interfere with antineoplastic drugs.
Lehouritis [81], 2015	*Escherichia coli* nitroreductase activity is able to enhance the cytotoxicity of the drug CB1954.
Iida [76], 2013	Shifts in microbiota decrease the production of ROS and oxidative damage, key mechanisms in many anticancer drugs.
Viaud [82], 2013	Gram-positive bacterial decontamination with antibiotics reduces the stimulation of the Th1 and Th17 immune responses, thus impairing the efficacy of cyclophosphamide.
Frank [83], 2015	Methotrexate can induce gastrointestinal toxicity: in murine models, gut microbiota depletion has been linked to poorer TLR2 activation and therefore lower expression of the multidrug resistance pump ABCB1/MDR1. The TLR2 pathway has been proven to reduce the toxic effects of methotrexate on the gut epithelium.

**Table 3:** Effects of different antibiotics on intestinal microbiota.

Antibiotic	Effects on Intestinal Microbial
Amoxicillin/clavulanate acid	Reduction in bacterial diversity Increase in abundance of *Enterococcus* spp. and *Enterobacteriaceae* (*Citrobacter* spp., *Klebsiella* spp., *Proteus* spp.) Reduction in *Clostridium* spp., *Bifidobacterium* spp., *Lactobacillus* spp., *Roseburia* spp.
Cephalosporins	Depletion of *Enterobacteriaceae* spp. and *Escherichia coli* Increase in the abundance of *Enterococcus*, *Citrobacter* spp., *Klebsiella* spp., *Pseudomonas* spp. Increase in the colonization of *Clostridium difficile* for high generation cephalosporins
Piperacillin	Increase in abundance of *Enterococcus* spp. and *Escherichia coli* Depletion of *Bacteroides* spp., *Bifidobacterium* spp., *Clostridium* spp., *Lactospirum* spp., *Lactobacillus* spp.
Trimethoprim-Sulfamethoxazole	Reduction in the abundance of *Enterobacteriaceae* spp. and *Escherichia coli* Increase of resistant *Escherichia coli*, *Acinetobacter* spp. and *Pseudomonas* spp.
Fluoroquinolones	Reduction in the abundance *of Enterobacteriaceae*, *Escherichia coli*, *Bacillus* spp., *Corynebacterium* spp. Depletion of some anaerobic bacteria (*Bacteroides* spp., *Bifidobacterium* spp., *Lactobacillus* spp., *Peptostreptococcus* spp., *Veilonella* spp.) Increase in the abundance of *Citrobacter* spp., *Enterobacter* and *Klebsiella* spp.
